# Using community health workers to assess the prevalence of dementia in a community based sample of older Zambians

**DOI:** 10.1002/alz.092555

**Published:** 2025-01-09

**Authors:** Mataa M Mataa, Lisa Kalungwana, Chiti Bwalya, Faith Simushi, Lottie Hachaambwa, Linah Mwango, Coolwe Namangala, Leroy Yankae, David Daniel, Jeremy A. Tanner, Melissa A Elafros

**Affiliations:** ^1^ Chipata Level One Hospital, Lusaka, Lusaka Zambia; ^2^ John Snow Health LTD, Lusaka, Lusaka Zambia; ^3^ University of Maryland, College Park, MD USA; ^4^ Maryland Global Initiatives Corporation, Lusaka, Lusaka Zambia; ^5^ University Teaching Hospitals, Lusaka, Lusaka Zambia; ^6^ Ciheb Zambia, Lusaka, Lusaka Zambia; ^7^ University of Maryland, Baltimore, MD USA; ^8^ Biggs Institute for Alzheimer’s and Neurodegenerative Disease, University of Texas Health San Antonio, San Antonio, TX USA; ^9^ University of Michigan, Ann Arbor, MI USA

## Abstract

**Background:**

In Zambia, dementia prevalence is unknown due to limited community awareness and a lack of providers skilled in recognizing and diagnosing this disease. Community healthcare workers (CHWs) are widely utilized across sub‐Saharan Africa to improve health care access, particularly HIV services. CHWs may be an untapped resource to raise awareness, screen for dementia, and support dementia care in the community. This study is piloting a community‐based model using CHWs to screen for dementia and associated risk factors in a population‐based sample of older Zambians.

**Methods:**

Ten Zambian CHWs underwent a five‐day training led by a neuropsychologist and neurologist on symptoms of dementia, myths, and administration of the 10/66 dementia screening protocol. CHWs were then deployed in two high‐density Lusaka communities to enroll adults age >55 and a caregiver from randomly selected households. Demographics, medical history, diet, anthropomorphic measurements, HIV, and glucose testing were collected. Dementia was defined using the CSI‐D with a neuropsychologist regularly assessing community administration to assure standardization

**Results:**

To date, 185 Zambians (mean age 67; 66% female, mean education 5.7yrs) have been enrolled. Mean household income was ZMW 1339 ($52/month). Fifty percent endorsed food insecurity in the past 30 days, whereas 25% drank alcohol. Twenty‐four (13%) participants were HIV‐positive. One hundred and two (55%) participants regularly attended community social activities. Caregivers reported that 13 (7%) participants regularly forgot family member’s names, 7 (4%) got lost in the community, and 5 (3%) got lost at home. On dementia screening, 63 (34%) screened negative for dementia (dfscore <0.12), 23 (12%) screened in the intermediate range (dfscore 0.12 to <0.184) and 99 (54%) participants screened positive for possible dementia (dfscore ≥0.184).

**Conclusions:**

CHWs can be trained to reliably administer dementia screening measures and may serve as a resource for dementia care. High rates of positive dementia screening raise concern that these measures may be overly sensitive in this population. We are conducting additional neurological evaluation and neurocognitive testing with participants to assess CSI‐D sensitivity and specificity and to determine the prevalence of dementia and dementia risk factors in Zambia.

